# Synergistic PD‐1/LAG‐3 Inhibition: Mechanistic Insights and Implications for Enhanced Tumor Immunotherapy

**DOI:** 10.1002/mco2.70216

**Published:** 2025-05-20

**Authors:** Jianqiao Shentu, Hening Xu, Shiwei Duan

**Affiliations:** ^1^ Department of Clinical Medicine School of Medicine, Hangzhou City University Hangzhou Zhejiang China

1

In the August issue of *Cell* 2024, three pivotal studies published in *Cell* by the Vignali and Wherry research teams advanced our understanding of programmed cell death protein 1 (PD‐1)/lymphocyte activation gene 3 (LAG‐3) synergy in T cell exhaustion [1, 2, 3]. This provides new therapeutic strategies for clinical guidance in anti‐infection and antitumor treatments.

PD‐1 and LAG‐3 are both key inhibitory receptors (IRs). In the context of tumors and chronic viral infections, their expression on CD8^+^ T cells increase, hampering the immune system's ability to eliminate these threats. Research indicates that PD‐1 and LAG‐3 jointly contribute to T cell exhaustion, diminishing effector functions and weakening antitumor immunity. Clinically, the combination of PD‐1 and LAG‐3 inhibitors has shown remarkable success—particularly in the RELATIVITY‐047 melanoma trial—improving progression‐free survival (PFS) and demonstrating superior efficacy in neoadjuvant settings compared to PD‐1 monotherapy, while maintaining a favorable toxicity profile. However, mechanistic uncertainties persist [4].

In the first study, the Vignali team utilized mouse models and clinical data to explore the synergistic effects of PD‐1 and LAG‐3 on T cell function and antitumor immunity [2]. In a melanoma mouse model, CD8^+^ T cells deficient in both PD‐1 and LAG‐3 exhibited enhanced tumor clearance and improved survival rates. Transcriptomic analysis revealed that the absence of PD‐1 and LAG‐3 led to increased T‐cell receptor (TCR) clonality and upregulation of effector‐like and interferon‐responsive genes. These transcriptional changes boosted IFN‐γ secretion and enhanced T cell antitumor efficacy. The study discovered that the increase in IFN‐γ does not directly drive antitumor immunity by regulating tumor cells or other cells within the tumor microenvironment. Instead, it acts on CD8^+^ T cells themselves in an intracellular and autocrine manner to enhance their function. Particularly, the upregulation of CCL5 may play a crucial role in this process, although CCL5 might have diverse functions in different environments. Further studies have demonstrated that PD‐1 and LAG‐3 jointly promote the exhaustion of CD8^+^ T cells and restrict their proliferation and effector functions. This process is closely associated with the regulation of the transcription factor TOX. Specifically, the loss of PD‐1 and LAG‐3 significantly reduces the expression level of TOX, indicating that these two receptors have a synergistic effect in regulating the development of T cell exhaustion. Especially in regulating TOX expression, LAG‐3 exhibits a more significant dominant role. Additionally, the study also found that in the context of PD‐1 and LAG‐3 loss, the expression pattern of natural killer group 2 member A (NKG2A) shows interesting inconsistencies in monoclonal and polyclonal systems. This difference suggests that the expression of NKG2A may affect TCR clonal expansion and antitumor immune response. NKG2A may play a key regulatory role in the synergistic effect of PD‐1 and LAG‐3, and blocking NKG2A could be a beneficial supplement to the combined treatment of PD‐1 and LAG‐3.

The second study (initiated by Vignali et al.), focused on the clinical combination of Relatlimab and Nivolumab in advanced melanoma, demonstrated that dual blockade enhanced receptor signaling in CD8^+^ T cells, altering their differentiation to improve cytotoxicity while retaining certain exhaustion characteristics [3]. The study revealed that the combination of Relatlimab and Nivolumab led to a unique state of CD8^+^ T cells, where enhanced cytotoxicity and exhaustion gene modules coexisted. Despite the presence of exhausted transcriptional signatures, the treatment significantly enhanced the cytotoxicity of CD8^+^ T cells, which might suggest that the inhibition of PD‐1 and LAG‐3 enhances short‐term immune responses at the expense of long‐term persistence. Further studies indicated that the coexpression of cytotoxic and exhaustion markers of CD8^+^ T cells was driven by PRDM1, BATF, ETV7, and TOX. The effector function of clonally expanded CD8^+^ T cells after treatment was significantly upregulated, and the characteristics of intratumoral CD8^+^ T cells were associated with a favorable prognosis. Notably, in peripheral blood, this marker of combined treatment with Relatlimab and Nivolumab was confirmed as an increased frequency of CD38^+^ TIM3^+^ CD8^+^ T cells. This finding may provide a precise method for identifying pharmacodynamic responses in patients in the clinic and assist in screening out patients who may be resistant to combined treatment with Relatlimab and Nivolumab.

In the third study, Wherry's team investigated the regulation of Tex cells by PD‐1 and LAG‐3 in chronic viral infections and cancer [1]. They found that LAG‐3 sustains TOX expression and modulates the CD94/NKG2‐Qa‐1b axis, influencing NK receptor expression and Tex cytotoxicity. LAG‐3 drives the formation of a CD94/NKG2^+^ Tex subset by recognizing the stress ligand Qa‐1b, with similar observations in human studies. This research highlights the distinct roles of PD‐1 and LAG‐3 in regulating T cell exhaustion, pointing to LAG‐3's role in maintaining Tex persistence and enhancing cytotoxicity.

Together, these studies uncover the mechanisms through which PD‐1 and LAG‐3 modulate CD8^+^ T cell function (Figure 1). Vignali's work emphasizes the balance between effector function and exhaustion, suggesting that LAG‐3 inhibition may enhance the immune response at the expense of long‐term T cell persistence. Wherry's findings complement this by highlighting LAG‐3's role in sustaining T cell cytotoxicity and persistence, providing a comprehensive view of the dual checkpoint blockade.

**FIGURE 1:**
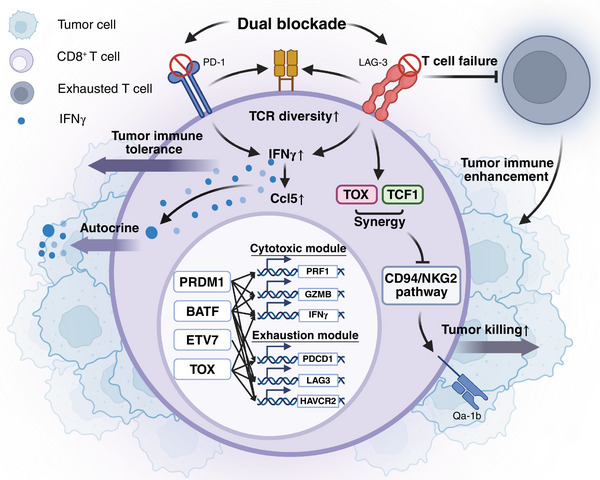
Synergistic effects of LAG‐3 and PD‐1 on CD8^+^ T cell tumor immunity. Lymphocyte activation gene 3 (LAG‐3) and programmed cell death protein 1 (PD‐1) synergistically inhibit CD8^+^ T cell function. Blocking both pathways enhances cytotoxicity and reduces exhaustion in CD8^+^ T cells, improving antitumor immunity. In the absence of LAG‐3 and PD‐1, CD8^+^ T cells demonstrate increased T‐cell receptor (TCR) diversity, IFN‐γ production, and Ccl5 gene expression, enhancing their ability to target tumors. Loss of LAG‐3 downregulates TCF1 and TOX, inhibiting the CD94/natural killer group 2 member A (NKG2) axis and boosting tumor‐killing activity via Qa‐1b ligands. These findings deepen the understanding of LAG‐3 and PD‐1 synergy and suggest new directions for tumor immunotherapy.

While these findings represent significant progress, there are limitations. In the clinical trial led by Vignali et al. [3], Relatlimab monotherapy lasted for only 4 weeks (all patient groups received dual antibody treatment after 4 weeks). As a result, it remains unclear whether the results observed after anti‐LAG‐3 treatment will persist. Second, the study's reliance on single‐cell sequencing presents a problem [3]. In some cases, single‐cell sequencing may not be able to capture cell‐to‐cell interactions and dynamic changes. Moreover, it mainly focuses on gene expression levels while ignoring many other important biological characteristics such as cell metabolic state, protein expression, and signaling pathway activity. Therefore, in the future, it might be necessary to combine multiple technical means, such as multiomics data integration, flow cytometry, and imaging analysis, to further verify its key findings. Additionally, in two other mouse studies, an important finding was made: the combined loss of PD‐1 and LAG‐3 led to the emergence of NK‐like effector CD8^+^ T cells, which are characterized by the simultaneous expression of activating and inhibitory NK receptors on the surface [1, 2]. It should be noted that given the differences between the mouse model and the human immune system in some key aspects, the verification of this finding depends on further human research in the future to provide a definitive answer.

Moreover, current clinical trials must expand to include broader patient populations and diverse tumor types to ensure the safety and efficacy of PD‐1 and LAG‐3 inhibitors across different contexts. Personalized treatment approaches, guided by biomarkers that predict patient responses, are essential for refining therapeutic regimens. A recent study demonstrated that LAG‐3 and PD‐1 inhibitors enhance outcomes in advanced nasopharyngeal carcinoma (NPC). Immunotherapy‐naïve patients showed 33.3% objective response rate (ORR), 75% disease control rate (DCR), and 10.8‐month median PFS, outperforming PD‐1 monotherapy. Responses persisted in resistant cases (11.8% ORR, 64.7% DCR). This dual blockade holds clinical promise, particularly as frontline therapy for advanced NPC [5].

In conclusion, these studies offer new insights into the regulation of immune checkpoints, providing a foundation for future research and clinical applications. They underscore the need for further experiments and trials to better understand the mechanisms of PD‐1 and LAG‐3, optimize immunotherapy strategies, and ultimately improve patient outcomes.

## Author Contributions

Jianqiao Shentu analyzed the literature, wrote the manuscript, Jianqiao Shentu and Hening Xu drafted Figure 1. Jianqiao Shentu and Shiwei Duan conceived the idea. Shiwei Duan reviewed and revised the manuscript. All the authors gave the final approval of the submitted version.

## Ethics Statement

Not applicable.

## Conflicts of Interest

The authors declare no conflicts of interest.

## Data Availability

Not applicable.
